# Baseline White Matter Is Associated With Physical Fitness Change in Preclinical Alzheimer’s Disease

**DOI:** 10.3389/fnagi.2020.00115

**Published:** 2020-04-29

**Authors:** Vijay K. Venkatraman, Christopher E. Steward, Kay L. Cox, Kathryn A. Ellis, Pramit M. Phal, Matthew J. Sharman, Victor L. Villemagne, Michelle M. Y. Lai, Elizabeth V. Cyarto, David Ames, Cassandra Szoeke, Christopher C. Rowe, Colin L. Masters, Nicola T. Lautenschlager, Patricia M. Desmond

**Affiliations:** ^1^Department of Medicine and Radiology, The University of Melbourne, Melbourne, VIC, Australia; ^2^Department of Radiology, Royal Melbourne Hospital, Melbourne, VIC, Australia; ^3^School of Medicine, University of Western Australia, Perth, WA, Australia; ^4^Academic Unit for Psychiatry of Old Age, Department of Psychiatry, The University of Melbourne, Melbourne, VIC, Australia; ^5^The Florey Institute of Neuroscience and Mental Health, The University of Melbourne, Melbourne, VIC, Australia; ^6^Melbourne School of Psychological Sciences, University of Melbourne, Melbourne, VIC, Australia; ^7^Epworth Medical Imaging, Richmond, VIC, Australia; ^8^School of Health Sciences, University of Tasmania, Launceston, TAS, Australia; ^9^Department of Molecular Imaging and Therapy, Austin Health, Melbourne, VIC, Australia; ^10^South Metropolitan Health Service, Perth, WA, Australia; ^11^Curtin Medical School, Curtin University, Perth, WA, Australia; ^12^Bolton Clarke Research Institute, Brisbane, QLD, Australia; ^13^National Ageing Research Institute, Melbourne, VIC, Australia; ^14^St George’s Hospital, Kew, VIC, Australia; ^15^Centre for Medical Research, Royal Melbourne Hospital, Melbourne, VIC, Australia; ^16^Healthy Brain Initiative, Australian Catholic University, Melbourne, VIC, Australia; ^17^Melbourne Dementia Research Centre, The University of Melbourne, Melbourne, VIC, Australia; ^18^NorthWestern Mental Health, Melbourne Health, Melbourne, VIC, Australia

**Keywords:** MCI (mild cognitive impairment), MRI, DWI, objective physical fitness measures, physical activity intervention

## Abstract

White matter (WM) microstructure is a sensitive marker to distinguish individuals at risk of Alzheimer’s disease. The association of objective physical fitness (PF) measures and WM microstructure has not been explored and mixed results reported with physical activity (PA). Longitudinal studies of WM with PA and PF measures have had limited investigation. This study explored the relationship between objective PF measures over 24-months with “normal-appearing” WM microstructure. Data acquired on magnetic resonance imaging was used to measure “normal-appearing” WM microstructure at baseline and 24-months. Clinical variables such as cognitive and blood-based measures were collected longitudinally. Also, as part of the randomized controlled trial of a PA, extensive measures of PA and fitness were obtained over the 24 months. Bilateral corticospinal tracts (CST) and the corpus callosum showed a significant association between PF performance over 24-months and baseline WM microstructural measures. There was no significant longitudinal effect of the intervention or PF performance over 24-months. Baseline WM microstructural measures were significantly associated with PF performance over 24-months in this cohort of participants with vascular risk factors and at risk of Alzheimer’s disease with distinctive patterns for each PF test.

## Introduction

Physical inactivity is estimated to be the third-largest modifiable risk factor in dementia, after limited formal education and smoking (Norton et al., 2014). In individuals with mild cognitive impairment (MCI) or subjective memory complaints (SMC) who are at increased risk of dementia, physical activity (PA) has been shown to improve cognition as well as increased fitness, function, mobility, and strength (Norton et al., 2014). PA interventions with ≥150 min/week of moderate-to-vigorous PA (Falck et al., 2017) have been investigated as a primary strategy to mitigate cognitive decline. Lower cardiovascular and motor fitness are associated with cognitive impairment in nondemented subjects examined at a memory clinic (Gnosa et al., 2019). Recent studies (Lautenschlager et al., 2008; Cox et al., 2019) have shown that long-term PA intervention adherence is achievable and has health benefits.

There is increasing evidence that interventions such as PA result in dynamic changes in white matter (WM) structure throughout the lifespan, highlighting the importance of WM plasticity (Sampaio-Baptista and Johansen-Berg, 2017; Wassenaar et al., 2019). Cross-sectional studies have shown associations between WM microstructure (WMM) measured using diffusion tensor (DT) imaging (with or without WM hyperintense lesions) and the amount of PA in older adults (Burzynska et al., 2014; Tian et al., 2015). Earlier studies reported that higher levels of physical fitness (PF) are associated with increased frontal and corpus-callosum WM volume (Erickson et al., 2007). However, a recent review found mixed results (Sexton et al., 2016) on cross-sectional associations between PA and WMM. Increased fractional anisotropy (FA) with higher levels of PF has been shown within the corpus-callosum (Johnson et al., 2012), superior longitudinal fasciculus and arcuate fasciculus (Liu et al., 2012), and superior/inferior longitudinal fasciculus, superior corona radiate (Tseng et al., 2013). In the studies that examined mean diffusivity (MD), two studies (Marks et al., 2011; Johnson et al., 2012) reported no significant finding. Another study reported on decreases in MD in non-overlapping regions to their FA results, within the cingulum and posterior thalamic radiation (Tseng et al., 2013). Only one study examined axial diffusivity (AxD) and radial diffusivity (RD; Johnson et al., 2012), finding that reductions in RD accompanied increases in FA. In people living with MCI compared with cognitively normal individuals, studies (Chua et al., 2008; Di Paola et al., 2010; Stricker et al., 2016) have shown reduced anisotropy in the corpus-callosum, temporal WM, and parietal WM over and above age-related changes in WMM). Researchers (Di Paola et al., 2010) propose two possible mechanisms to account for the corpus-callosum changes: (a) with increased AxD without a change in FA (Wallerian degeneration); and (b) decreased FA increased RD without AxD change (Retrogenesis). In SMC participants, researchers (Ohlhauser et al., 2019) have been shown widespread changes in WM with decreased FA, decreased MD, and increased RD compared with cognitively normal participants.

Vascular risk factors in dementia have been shown to contribute to impairments observed in the pre-clinical stage of cognitive decline. Vascular risk factors have been associated with reduced WMM among older adults and predicted faster cognitive decline. The detrimental effects of vascular risk factors on WMM have also been shown to be exacerbated among APOE ε4 carriers (Power et al., 2017). Vascular risk factors such as arterial hypertension, blood pressure, and elevated total cholesterol level have shown to play significant roles in the development of vascular MCI (Stephan et al., 2009; Meguro and Dodge, 2019). Accumulation of multiple vascular risk factors has shown to affect longitudinal WMM integrity in patients with MCI and dementia (Maillard et al., 2015).

Longitudinal studies exploring the relationship of WMM with PA are limited. 1 study with a 1-year intervention did not report any significant WMM (FA, MD, AxD, RD measures) change (Voss et al., 2013). For global WMM, higher levels of PA were associated with increased FA at 3-year follow-up, with no significant relationships for diffusivity values (Gow et al., 2012).

Our earlier report demonstrated that a home-based, long-term (24-months) moderate-intensity PA intervention is effective in increasing PF outcome measures in the individuals at risk of Alzheimer’s disease with vascular risk factors (Cox et al., 2019). However, we have reported that PA intervention (Venkatraman et al., 2020) did not delay the progression of WM lesions or hippocampal volume loss in older adults with SMC or MCI and vascular risk factors. These results suggest that there is a need for personalized PA programs that are designed the biological and genetic moderators of exercise efficacy (Barha et al., 2017; Walsh and Tschakovsky, 2018; You et al., 2019). A recent study (Opel et al., 2019) explored the association between PF and cognition in healthy, young adults and showed that higher FA was significantly associated with enhanced global cognitive function and FA was shown to significantly mediate the association between walking endurance and cognitive performance. This study also highlighted the importance of investigating the effects of PF on WMM in longitudinal and interventional studies.

To our knowledge, there is currently no study in individuals with vascular risk factors and risk of Alzheimer’s disease that has reported on effects of a long-term (24-months) PA intervention on longitudinal WMM or explored the relationship between objective outcome measures of PF over 24-months with WMM (cross-sectional and longitudinal).

In this study, two major motor WM tracts were explored: (i) the corpus-callosum—which integrates cognitive, motor, and sensory information between the left and right hemispheres of the brain; and (ii) the bilateral corticospinal tracts (CST)—the only descending motor tract that is known to make monosynaptic connections with spinal motoneurons. In this study, we explored the effect of a moderate 24-month PA intervention on longitudinal WMM in individuals with vascular risk factors at risk of Alzheimer’s disease. We also examined the association between baseline and longitudinal WMM measures with a change in PF measures over 24 months [explored as group differences between those participants who improved their PF (improvers) and those who did not (decliners)].

## Materials and Methods

### Participants

The Australian Imaging, Biomarkers, and Lifestyle (AIBL) Active study explored the predictive value of biomarkers, cognitive variables and lifestyle factors for future progression to Alzheimer’s disease (Cyarto et al., 2012; Cox et al., 2019). Community-dwelling older adults were recruited in the Melbourne metropolitan area from the participants of the AIBL flagship study (Ellis et al., 2009). AIBL is a multi-disciplinary prospective longitudinal study of aging which follows (one thousand one hundred twelve) older volunteers who live in Melbourne and Perth.

The AIBL Active sample consisted of 108 older volunteers living in Melbourne, Australia (median age—73 years, interquartile range—7 years) with at least one vascular risk factor recruited from the AIBL flagship study. Inclusion criteria include: (i) aged 60 years or over at last birthday; (ii) diagnosis of SMC or MCI; (iii) community-dwelling; (iv) presence of at least one vascular risk factor (such as obesity, hypertension, heart disease, type II diabetes, smoking, hypercholesterolemia and doing less than 150 min/week of moderate PA); and (v) understands written and spoken English. Exclusion criteria include: (i) baseline Standardized Mini-Mental State Examination score (SMMSE) <24 (Thurfjell et al., 2014) or diagnosis of dementia; (ii) unable to have MRI scans; (iii) limited mobility (e.g., unable to walk or require a walking aid for balance); (iv) show evidence of pervasive depression; (v) current history of alcohol dependance; (vi) unstable or life-threatening medical condition; (vii) medical condition that contra-indicates PA; (viii) severe visual or hearing impairment; (ix) unable to attend the follow-up visits; or (x) participating in another randomized controlled trial. The study was approved by the Melbourne Health Human Research Ethics Committee. In this article, we report results for the 79 participants who had a diffusion magnetic resonance (MR) scan out of the 108 participants and the PF data at both baseline and 24-months.

### Physical Activity Intervention

The intervention protocol is explained in detail in our previous reports (Cyarto et al., 2012; Cox et al., 2019) and briefly summarized here. The intervention package comprised three components; the PA program, the behavioral intervention package, and phone monitoring. The 24-month intervention period was divided into four stages. Stage 1: 0–6 months; Stage 2: 6–12 months; Stage 3: 12–18 months; Stage 4: 18–24 months. Intervention participants attended an individual PA workshop within 2–4 weeks of their baseline visit. During this 60-min session, the program manual was given to participants and they received instructions on their PA program, recording and the use of the behavioral intervention material. The PA program was individualized for each participant by the addition of minutes of moderate PA to their baseline habitual PA with a final target of at least 150 min/week of moderate-intensity PA. The PA started slowly and progressed gradually taking 8 weeks to reach the target amount and intensity. Educational material and recommendations for a healthy lifestyle (excluding PA information), were given to both groups. Intervention participants received a manual containing the PA program and the behavioral intervention using workshops and with 17 newsletters containing additional motivating material mailed at regular intervals. The 15-min standardized and structured calls were used to monitor and give feedback on the participant’s progress and encourage their continuing adherence. The control group continued with their usual PA for the 24-month study period. In addition to the educational material, the control group (usual care) participants received newsletters containing generic non-PA information and were contacted by phone at the same frequency as the intervention group with conversation limited to everyday topics with no discussion about PA. This was to ensure that the control and intervention group had similar study contact. The control group was offered a PA workshop at the end of the study.

### Physical Fitness Measures

Each participant was assigned to the intervention or control group as part of the randomized control trial. For each participant, their PA was measured using a pedometer and the Community Healthy Activities Model Program for Seniors PA questionnaire (Stewart et al., 2001) for older adults. The pedometer provides an objective measure of ambulatory PA. Participants wore a pedometer (DigiWalker SW-200, Yamax Inc., Tokyo, Japan) during their usual activities during the week for five weekdays and one weekend.

The following PF measures at baseline and 24-months were used in this study: (a) 6-min Walk Test: this test assesses cardiovascular fitness, with the participant walking a standardized course for 6 min and measuring the distance in meters. (b) Sit-to-Stand Test: A test of functional lower limb or leg strength. The participant sits in a standard chair and stands up and down five times as quickly as possible while being timed. (c) Timed Up and Go Test (TUG): the participant is timed in seconds while standing up from a standard chair, walking 3 m and then returning to sit again in the chair. The TUG assesses agility and mobility. (d) Grip strength: measured in kilograms (kg) using a hand-grip dynamometer on both dominant and non-dominant hands. Percent change of PF measures were used to categorize the participants (Mason et al., 2016) as improvers (≥10%) and decliners (≤-10%) for each PF measure.

### MR Imaging Acquisition

Brain imaging scans at baseline were obtained at The Royal Melbourne Hospital on a 3-Tesla Siemens Tim Trio MR scanner, 12-channel head array coil. Each participant acquired structural T_1_-weighted Magnetization Prepared Rapid Gradient Echo images (TR/TE/TI = 1,900/2.13/900 ms, FOV = 256 × 256 mm, matrix = 256 × 256, flip angle = 9°, isotropic 1-mm voxel), Fluid-attenuated inversion recovery images (TR/TE/TI = 5,000/355/1,800 ms, FOV = 256 × 256 mm, matrix 256 × 256, flip angle = 120°, voxel size = 0.5 × 0.5 × 1) and diffusion-weighted imaging (single-SE diffusion-weighted EPI with TR/TE = 8,700/92 ms, FOV = 240 × 240 mm, matrix = 96 × 96, *b* = 1,000, voxel size 2.5 mm^3^, 30 directions). Participants were also scanned at 24-months.

### Aβ-Amyloid Imaging Acquisition and Analysis

Seventy-two participants (F-18 Florbetapir, *n* = 63; F-18 Flutemetamol, *n* = 8; F-18 Florbetaben, *n* = 1) had baseline Aβ-amyloid imaging. The computed cortical standardized uptake value ratio (SUVR) was considered as a dichotomous variable (high = Aβ+ vs. low = Aβ−). Here, participants were classified as being Aβ+ when the SUVR was ≥1.10 for florbetapir (Clark et al., 2011), SUVR ≥0.62 for flutemetamol (Thurfjell et al., 2014) was ≥1.45 for florbetaben and ≥1.50 for PiB (Villemagne et al., 2011).

### Diffusion Image Analysis

Diffusion MR imaging data for baseline and 24-months were pre-processed using the Tortoise software package v3.1.1 (Pierpaoli et al., 2010; Irfanoglu et al., 2017). The raw diffusion images were AC-PC corrected and deskulled using AFNI (Cox, 1996), corrected for subject motion, eddy-current artifacts and EPI susceptibility distortions using DIFFPREP (Pierpaoli et al., 2010; Irfanoglu et al., 2017). The data was then used to create a study-specific, unbiased, tensor template with DTI-TK (Keihaninejad et al., 2012). This template was integrated with FSL TBSS (Smith et al., 2006) to generate 4D DT images for all participants. WM hyperintense lesions were manually segmented from the baseline FLAIR images and checked by two qualified neuroradiologists (PP and PD). A Lesion probability map (LPM) was generated from all 79 participants and ≤5% voxels were excluded from the analysis to restrict the diffusion measures within “normal-appearing” WM (Bisecco et al., 2016). The longitudinal diffusion measures were calculated as a percent change of baseline and 24-months.

### Other Variables of Interest

Participants had comprehensive longitudinal assessments comprising of cognitive measures, body mass index, waist circumference, and a fasting blood sample (Cyarto et al., 2012). Presence of at least one APOE ε4 allele was determined from the baseline blood sample. The Consortium to Establish a Registry for Alzheimer’s Disease neuropsychological assessment battery was used for identifying MCI (Welsh et al., 1994). The Memory Complaint Questionnaire score (Crook et al., 1992) was used to determine perceived cognitive decline (SMC). Vascular risk factors were converted into categorical variables for physical inactivity, obesity, hypertension, type II diabetes, smoking, hypercholesterolemia, and cerebrovascular disease. The following well-established thresholds were used for the factors: physical inactivity (<150 min/week of moderate PA), obesity (body mass index >25 kg/m^2^, waist circumference >94 cm for male and >80 cm for female), hypertension (systolic and diastolic blood pressure ≥140/90 mm Hg), self-reported medication, self-reported hypertension, type II diabetes [baseline fasting glucose (≥7 mmol/l), self-reported diabetes, self-reported medications], smoking (current smoker, smoker in the last 1 year), and hypercholesterolemia (total cholesterol >6.22 mmol/l, triglycerides >2.26 mmol/l, self-reported high cholesterol, self-reported medication) and self-reported cerebrovascular disease.

### Statistical Methods

A comparison between those who were recruited in this study (*n* = 108) and those who had DTI scans with longitudinal PF measures (*n* = 79) was performed to detect any selection bias. Sample sizes and percentages are reported for categorical variables and groups were compared using Fisher’s exact chi-square test. The continuous variables were tested for normality using the Shapiro–Wilk test, for normally distributed data the groups were compared using independent samples *t*-test otherwise using the Mann–Whitney U test.

The dichotomous vascular risk variables were compared between baseline and 24-month measures using McNemar’s test. The cognitive status (normal, SMC or MCI) was compared between baseline and 24-month using Friedman’s test. The WM hyperintense lesion volume (a measure of WM disease) was compared between baseline and 24-month using the Mann–Whitney U test.

FSL Randomise was used to examine the voxel-wise group differences in FA, RD, MD, and AxD maps adjusted for age, gender, years of education, APOE ε4 status and cognitive status. The results were corrected for multiple comparison correction using Family-Wise Error (FWE; *p* < 0.05) and obtained by performing voxel-wise statistics using Threshold-Free Cluster Enhancement. To identify the area of significant correlation, the study-specific template was registered to the FSL FMRIB58-FA_1 mm image using ANTS (Avants et al., 2008), and the Juelich Histological Atlas (Eickhoff et al., 2007) used to identify the CST and corpus callosum (CC).

First, we compared the longitudinal WMM measures (FA, MD, RD, and AxD maps) between the intervention and control groups adjusted for age, gender, years of education, APOE ε4 status and cognitive status.

Second, we evaluated the association of baseline WMM measures between the fitness improvers and decliner groups adjusted for age, gender, years of education, APOE ε4 status and cognitive status. The analysis was repeated by using intervention/control grouping, Aβ-amyloid status, and individual vascular risk factors such as hypertension status, obesity, smoking status, diabetes status, cholesterol status, physical inactivity status and cerebrovascular status as separate covariates in the model.

Third, we evaluated the association of change in WMM between the improvers and decliners defined by fitness measures adjusted for age, gender, years of education, APOE ε4 status and cognitive status.

## Results

Baseline demographic characteristics were not significantly (*p* > 0.05) different between our sample of 79 participants (who had a diffusion MR scan at baseline and longitudinal PF data) and the 108 participants who were recruited into the randomized control trial (Table 1). In the PA measures for all the participants over the 24 months, total steps/week declined by 0.31 ± 47.82%, and minutes and calories per week of moderate, high, and very high-intensity PA increased by 107.65 ± 298.28 and 90.22 ± 213.81%, respectively (Table 2).

**Table 1:** Baseline demographics characteristics of participants included in the study.

	Trial data (*N* = 108)	Our sample (*N* = 79)	Test statistics
Age in years^#^	73 (7)	72 (8)	*U* = 3,955.5, *p* = 0.40
Educational level in years^#^	15 (6)	15 (6)	*U* = 3,972.5, *p* = 0.42
Treatment Group (Intervention/Control)^∧^	55/53	39/40	*χ*^2^ = 0.04, *p* = 0.83
Female^∧^	57 (52.8%)	45 (57.0%)	*χ*^2^ = 0.32, *p* = 0.57
Current Smoker^∧^	4 (3.7%)	3 (3.8%)	*χ*^2^ = 0.001, *p* = 0.97
ApolipoproteinE ε4 carrier^∧^	29 (26.9%)	21 (26.6%)	*χ*^2^ = 0.002, *p* = 0.97
Mild Cognitively Impaired (Amnestic/Non-Amnestic)^∧^	31 (24/7)	20 (13/7)	*χ*^2^ = 0.95, *p* = 0.82
Cognitively Normal/Subjective memory complaints^∧^	15/62	14/45	*χ*^2^ = 0.63, *p* = 0.73
Amyloid Positive^∧^	23 (24.47%)	15 (20.8%)	*χ*^2^ = 0.31, *p* = 0.58
Alzheimer’s Disease Assessment Scale-Cognitive Subscale (ADAS Cog 11)^#^	7 (4)	6 (4)	*U* = 3,949.5, *p* = 0.38
Mini-Mental State Examination (0–30)^ #^	29 (2)	29 (1)	*U* = 4,216.5, *p* = 0.89
Geriatric Depression Scale Score (>3)^ #^	7	5	*U* = 4,220.0, *p* = 0.90
Body Mass Index (kg/m^2^)^ #^	26.6 (4.8)	26.5 (4.9)	*U* = 4,215.0, *p* = 0.89
Total Cholesterol (mmol/l)*	5.2 (1.1)	5.2 (1.0)	*t* = 0.074; *df* = 170.4; *p* = 0.94
Fasting Glucose (mmol/l)^ #^	5.1 (0.8)	5.1 (0.8)	*U* = 4,131.5; *p* = 0.99
Systolic Blood Pressure (mm Hg)*	126.3 (15.7)	125.8 (16.0)	*t* = −0.187; *df* = 165.54; *p* = 0.85
Diastolic Blood Pressure (mm Hg)*	72.1 (8.5)	71.8 (9.1)	*t* = −0.263; *df* = 161.98; *p* = 0.79
Brain-derived neurotrophic factor (BDNF, ng/ml)^#^	331.1 (477.1)	356.2 (524.4)	*U* = 3,969.5; *p* = 0.75
6-min walk test (m)*	492.6 (92.6)	499.3 (85.6)	*t* = 0.527; *df* = 1,74.93; *p* = 0.60
Timed up and go test (s)^#^	6.2 (2.3)	6.3 (2.3)	*U* = 4,259.0; *p* = 0.99
Sit-to-stand Time (s)^#^	10.7 (3.4)	10.8 (3.6)	*U* = 4,243.0; *p* = 0.95
Grip strength dominant hand (kg)*	32.3 (9.2)	31.8 (8.7)	*t* = −0.383; *df* = 172.60; *p* = 0.70
Grip strength Non-dominant hand (kg)*	30.1 (9.0)	29.3 (8.9)	*t* = −0.565; *df* = 168.87; *p* = 0.57

^#^Reported as Median (Interquartile Range). *Reported as Mean (Standard Deviation). ^∧^Reported as number of participants (Percentage) or number of participants.

**Table 2:** Percent change over 24-months for physical fitness and physical activity measures.

	All participants (*N* = 79)	Improvers	Decliners
**Physical Fitness Measures (percent change over 24-months)**			
6-min walk test (m)*	2.58 (16.71)	19.90 (11.71) *N* = 25	−19.19 (14.48) *N* = 14
Timed up and go test (s)*	3.57 (21.59)	−18.50 (6.38) *N* = 25	26.34 (16.79) *N* = 28
Sit-to-stand Time (s)*	−0.94 (19.92)	−22.22 (10.74) *N* = 26	22.38 (8.88) *N* = 25
Grip strength dominant hand (kg)*	−4.90 (14.89)	25.17 (10.36) *N* = 10	−17.41 (6.23) *N* = 32
Grip strength Non-dominant hand (kg)*	−7.83 (16.73)	20.94 (20.97) *N* = 8	−18.09 (5.59) *N* = 40
**Physical Activity Measures (percent change over 24-months)**			
Total steps/week*	−0.31 (47.82)	−	−
Minutes/week of medium, high and very high physical activity*	107.65 (298.28)	−	−
Calories/week of medium, high and very high physical activity*	90.22 (213.81)	−	−

*Reported as Mean (Standard Deviation).

The vascular risk factors (smoking, obesity, cerebrovascular, hypertension, cholesterol, and diabetes status) were not statistically different in our sample between baseline and 24-month period (Table 3). There is a significant change (improvement) in the PA status over 24-month, which is to be expected (Table 3). There was no statistical difference in WM hyperintense lesion volume over 24-month (Table 3).

**Table 3:** Summary of vascular risk factors and clinical characteristics at baseline and 24-months for 79 participants.

	Baseline	24-month	*p*-value
Physical activity intervention group*	39 (49.4%)	−	−
White matter lesion volume (mm^3^)^#^	3,591 (4,030)	5,207 (1,2778)	*U* = 2,619.0; *p* = 0.08
Cognitive status (Cognitively Normal/Subjective memory complaints/Mild Cognitively impaired)*	14/45/20	22/44/13	*N* = 79, *χ*^2^ = 3.67, *p* = 0.06
Smoking status*	30 (38%)	30 (38%)	*N* = 79, *p* = 1.00
Obesity status*	55 (69.6%)	50 (63.3%)	*N* = 79, *p* = 0.23
Cerebrovascular status*	7 (8.9%)	8 (10.1%)	*N* = 79, *p* = 1.00
Hypertension status*	44 (55.7%)	44 (55.7%)	*N* = 79, *p* = 1.00
Cholesterol status*	47 (59.5%)	44 (55.7%)	*N* = 79, *p* = 0.61
Diabetes status*	9 (11.4%)	10 (12.7%)	*N* = 79, *p* = 1.00
Physical Inactivity status*	35 (44.3%)	17 (21.5%)	*N* = 79, *p* = 0.03

^#^Reported as Median (Interquartile Range). *Reported as number of participants (Percentage).

### Group Difference in Longitudinal WMM: Intervention vs. Control

There were no statistically significant differences in longitudinal WMM measures of CST (left CST: *p* = 0.59 and right CST: *p* = 0.57 for FA; left CST: *p* = 0.37 and right CST: *p* = 0.64 for MD) and corpus-callosum (*p* = 0.50 for FA ; *p* = 0.85 for MD) between the intervention and control group (Table 4).

**Table 4:** Comparison of mean changes in diffusion metrics (fractional anisotropy and mean diffusivity) over time between intervention and usual-care (control) groups.

	Usual care group (Control)	Intervention group	*p*-values (FWE-corrected)
**Change in fractional anisotropy**			
Left CST	0.125 (0.112)	0.094 (0.027)	*p* = 0.586
Right CST	0.120 (0.111)	0.090 (0.024)	*p* = 0.574
CC	0.188 (0.063)	0.146 (0.132)	*p* = 0.497
**Change in mean diffusivity (10^−3^mm^2^s^−1^)**			
Left CST	0.334 (0.358)	0.221 (0.091)	*p* = 0.374
Right CST	0.295 (0.318)	0.217 (0.121)	*p* = 0.642
CC	0.890 (0.936)	0.665 (0.585)	*p* = 0.851

*The model was controlled for age, gender, years of education, APOE4, and baseline cognitive status. CC, corpus callosum; CST, corticospinal tract*.

### Change in PF Measures Over 24-Months: Improvers vs. Decliners

For the PF measures (Table 2), their percent change over 24-months was used to categorize the participants as improvers (≥10%) and decliners (≥−10%). For the 6-min walk test, the overall improvement was 2.58% over the 24 months. The change in the 6-min walk test was 19.90% for the improvers and −19.19% for the decliners. In the TUG test, the improvers showed improvement (completed the test faster) by 18.50% and decliners reduced by 26.34%. For the TUG test, the average of all the participants showed a decline of 3.57%. For the sit-to-stand test, the average change showed improvement (completed the test faster) by 0.94% with improvers showing 22.22% change and decliners −22.38% change. For the grip strength tests (dominant and non-dominant hand), both tests show an average decline over 24-months (4.90% and 7.83%). For the improvers in the grip strength tests, the change was 25.17% (dominant) and 20.94% (non-dominant). Table 5 shows the distribution of the baseline vascular risk factors between improvers and decliners within each subgroup.

**Table 5:** Baseline vascular risk factors for improvers and decliners in physical fitness performance, reported as percentage of subgroup.

	6-min walk test		Sit-to-Stand test		TUG test		Grip test (Dominant hand)		Grip test (Non-dominant hand)	
	Improvers (*N* = 25)	Decliners (*N* = 14)	Improvers (*N* = 26)	Decliners (*N* = 25)	Improvers (*N* = 25)	Decliners (*N* = 28)	Improvers (*N* = 10)	Decliners (*N* = 32)	Improvers (*N* = 8)	Decliners (*N* = 40)
Physical activity intervention group	60%	21.4%	69.2%	24%	60%	28.6%	40%	50%	25%	50%
Mild cognitively impaired	35.7%	28%	30.8%	20%	24%	28.6%	30%	34.4%	25%	22.5%
Smoking status	42.9%	28%	23.1%	56%	36%	46.4%	30%	28.1%	50%	37.5%
Obesity status	68%	71.4%	69.2%	68%	80%	64.3%	90%	75%	100%	67.5%
Cerebrovascular status	0%	4%	11.5%	4%	8%	10.7%	10%	12.5%	50%	7.5%
Hypertension status	42.9%	64%	53.9%	56%	56%	53.6%	40%	59.4%	50%	62.5%
Cholesterol status	64.3%	48%	57.7%	60%	60%	57.1%	70%	53.1%	87.5%	57.5%
Diabetes status	14.3%	8%	15.4%	4%	8%	14.3%	20%	15.6%	25%	12.5%
Physical Inactivity status	24%	64%	26.9%	52%	28%	46.4%	30%	40.6%	25%	45%

### Baseline WMM: Improvers vs. Decliners

#### 6-Min Walk Test (Cardiovascular Fitness)

**Figure 1:**
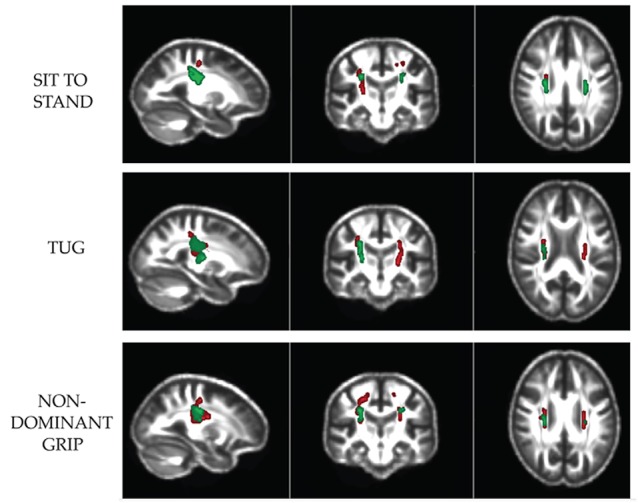
Axial, coronal and sagittal views of the significant regions (FWE *p* < 0.05) in the corticospinal tracts where baseline Axial Diffusivity (Green) and Fractional Anisotropy (Red) is associated with physical fitness improvement or decline over 24 months.

All diffusion measures of the three WM tracts showed no significant difference between the improvers vs. decliners (Table 6, Figure 1).

**Table 6:** Significant (FWE *p* < 0.05) white matter tracts for comparison of improvers vs. decliners in physical fitness (CC, corpus callosum; CST, corticospinal tract).

	6-min walk test (m)	Timed up and go test (s)	Sit-to-stand Time (s)	Grip strength dominant hand (kg)	Grip strength Non-dominant hand (kg)
**Fractional anisotropy**		*Left CST* **Improvers**: 0.548 (0.153) **Decliners**: 0.529 (0.152) **Cluster size: 373;** ***p* = 0.02** *Right CST* **Improvers**: 0.545 (0.150) **Decliners**: 0.522 (0.149) **Cluster size: 513;** ***p* = 0.045**	*Left CST* **Improvers**: 0.546 (0.155) **Decliners**: 0.529 (0.152) **Cluster size: 261**; ***p* = 0.032** *Right CST* **Improvers**: 0.540 (0.151) **Decliners**: 0.523 (0.146) **Cluster size: 585;** ***p* = 0.011**	*Left CST* **Improvers**: 0.541 (0.154) **Decliners**: 0.520 (0.159) **Cluster size: 80;** ***p* = 0.039**	*Left CST* **Improvers**: 0.540 (0.152) **Decliners**: 0.506 (0.147 **Cluster size: 393;** ***p* = 0.0005** *Right CST* **Improvers**: 0.536 (0.150) **Decliners**: 0.501 (0.147) **Cluster size: 518;** ***p* = 0.004**
**Mean diffusivity** (10^−3^ × mm^2.^s^−1^)	−	CC **Improvers**: 2.355 (0.529) **Decliners**: 2.404(0.521) **Cluster size: 320;** ***p* = 0.025**	−	−	−
**Radial diffusivity** (10^−3^ × mm^2.^s^−1^)	−	−	−	−	*Left CST* **Improvers**: 0.443 (0.140) **Decliners**: 0.478 (0.155) **Cluster size: 181;** ***p* = 0.004** *Right CST* **Improvers**: 0.450 (0.148) **Decliners**: 0.484 (0.189) **Cluster size: 349;** ***p* = 0.042**
**Axial diffusivity** (10^−3^ × mm^2.^s^−1^)	−	*Right CST* **Improvers**: 1.101 (0.249) **Decliners**: 1.085 (0.253) **Cluster size: 355;** ***p* = 0.0075**	*Left CST* **Improvers**: 1.087 (0.221) **Decliners**: 1.085 (0.226) **Cluster size: 269;** ***p* = 0.0122** *Right CST* **Improvers**: 1.101 (0.246) **Decliners**: 1.098 (0.251) **Cluster size: 150;** ***p* = 0.017** *CC* **Improvers**: 1.457 (0.305) **Decliners**: 1.462 (0.319) **Cluster size: 1747;** ***p* = 0.017**	*Right CST* **Improvers**: 1.097 (0.247) **Decliners**: 1.089 (0.252) **Cluster size: 349;** ***p* = 0.034**	*Right CST* **Improvers**: 1.103 (0.264) **Decliners**: 1.103 (0.247) **Cluster size: 191;** ***p* = 0.033**

#### TUG Test (Mobility Test)

**Figure 2:**
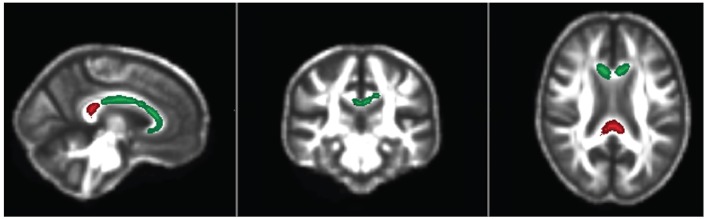
Axial, coronal and sagittal views of significant regions (FWE *p* < 0.05) in the corpuscallosum where baseline Axial Diffusivity (Green; Sit-to-stand test) and Mean Diffusivity (Red; TUG test) associated with physical fitness improvement or decline over 24 months.

FA was significantly different in left and right CST (FWE *p* = 0.02 and *p* = 0.045, respectively), corpus-callosum in MD (*p* = 0.025) and right CST (*p* = 0.008) in AxD (Table 6, Figures 1, 2).

#### Sit-to-Stand Test (Leg Strength Test)

FA and AxD was significantly different in left (*p* = 0.032 and 0.01 respectively) and right CST (*p* = 0.01 and 0.02, respectively); but also, AxD in the corpus-callosum (*p* = 0.017; Table 6, Figures 1, 2).

#### Grip Test (Dominant and Non-dominant; Hand Strength Tests)

There were group differences in FA *p* = 0.04 for the dominant hand in the left CST, and *p* < 0.001 for non-dominant in left CST; *p* = 0.004 for non-dominant in right CST, RD *p* = 0.004 for non-dominant in left CST, *p* = 0.04 for non-dominant in right CST and AxD *p* = 0.03 for dominant and non-dominant in right CST (Table 6, Figure 1).

In the FSL Randomise, none of the other variables (*p* > 0.05) such as the intervention/control grouping, Aβ-amyloid status, and cardiovascular risk factors altered the group difference between improvers and decliners of PF.

### Longitudinal WMM: Improvers vs. Decliners

There were no significant differences in longitudinal WMM measures (changes in WMM) over 24-months between the improvers and decliners in any of the PF tests. For CST (left and right) and CC, the change in FA (*p* = 0.29–0.86) and change in MD (*p* = 0.37–0.96) were not significantly different in any of the PF tests (Table 7).

**Table 7:** Mean changes in diffusion measures (longitudinal) over time for the various physical fitness tests.

	6-min walk test (metres)	Timed up and go test (seconds)	Sit-to-stand Time (seconds)	Grip strength dominant hand (kg)	Grip strength Non-dominant hand (kg)
**Change in fractional anisotropy**	*Left CST* **Improvers**: 0.114 (0.111) **Decliners**: 0.104 (0.031) (***p* = 0.856**) *Right CST* **Improvers**: 0.107 (0.111) **Decliners**: 0.102 (0.0275) (***p* = 0.678)** *CC* **Improvers**: 0.176 (0.131) **Decliners**: 0.158 (0.068)(***p* = 0.821**)	*Left CST* **Improvers**: 0.123 (0.117) **Decliners**: 0.098 (0.029) (***p* = 0.677**) *Right CST* **Improvers**: 0.116 (0.117) **Decliners**: 0.095 (0.025) (***p* = 0.842**) *CC* **Improvers**: 0.196 (0.139) **Decliners**: 0.143 (0.054) (***p* = 0.296**)	*Left CST* **Improvers**: 0.114 (0.106) **Decliners**: 0.103 (0.029) (***p* = 0.572**) *Right CST* **Improvers**: 0.109 (0.106) **Decliners**: 0.099 (0.025) (***p* = 0.625**) *CC* **Improvers**: 0.172 (0.086) **Decliners**: 0.161 (0.119) (***p* = 0.458**)	*Left CST* **Improvers**: 0.131 (0.153) **Decliners**: 0.102 (0.032) (***p* = 0.852**) *Right CST* **Improvers**: 0.123 (0.154) **Decliners**: 0.099 (0.028) (***p* = 0.845**) *CC* **Improvers**: 0.197 (0.159) **Decliners**: 0.157 (0.077) (***p* = 0.707**)	*Left CST* **Improvers**: 0.091 (0.031) **Decliners**: 0.115 (0.0918) (***p* = 0.693**) *Right CST* **Improvers**: 0.084 (0.036) **Decliners**: 0.111 (0.090) (***p* = 0.566**) *CC* **Improvers**: 0.142 (0.059) **Decliners**: 0.175 (0.114) (***p* = 0.608**)
**Change in mean diffusivity** (10^−3^ × mm^2.^s^−1^)	−*Left CST* **Improvers**: 0.295 (0.356) **Decliners**: 0.260 (0.113) (***p* = 0.783**) *Right CST* **Improvers**:newline 0.127 (0.326) **Decliners**: 0.240 (0.098) (***p* = 0.465**) *CC* **Improvers**: 0.862 (0.929) **Decliners**: 0.688 (0.594) (***p* = 0.677**)	*Left CST* **Improvers**: 0.328 (0.372) **Decliners**: 0.234 (0.099) (***p* = 0.625**) *Right CST* **Improvers**: 0.291 (0.333) **Decliners**: 0.226 (0.114)(***p* = 0.972**) *CC* **Improvers**: 1.0003 (1.05) **Decliners**: 0.581 (0.334) (***p* = 0.279**)	−*Left CST* **Improvers**: 0.301 (0.335) **Decliners**: 0.245 (0.119) (***p* = 0.380**) *Right CST* **Improvers**: 0.277 (0.312) **Decliners**: 0.227 (0.079)(***p* = 0.468**) *CC* **Improvers**: 0.769 (0.776) **Decliners**: 0.792 (0.811) (***p* = 0.856**)	−*Left CST* **Improvers**: 0.373 (0.476) **Decliners**: 0.244 (0.116) ***(p* = 0.745)** *Right CST* **Improvers**: 0.334 (0.437) **Decliners**: 0.228 (0.105) (***p* = 0.922**) *CC* **Improvers**: 0.690 (0.672) **Decliners**: 1.023 (1.018) (***p* = 0.534**)	−*Left CST* **Improvers**: 0.247 (0.084) **Decliners**: 0.287 (0.298) (***p* = 0.958**) *Right CST* **Improvers**: 0.199 (0.071) **Decliners**: 0.272 (0.270) (***p* = 0.374**) *CC* **Improvers**: 0.674 (0.570) **Decliners**: 0.807 (0.837) (***p* = 0.844**)

*The model was controlled for age, gender, years of education, APOE4 and cognitive status. CC, corpus callosum; CST, corticospinal tract*.

## Discussion

In this study of participants with vascular risk factors and at risk of dementia, we found three important results–(i) no significant effect of moderate PA intervention over 24-months on the “normal-appearing” WMM of the bilateral CST and corpus callosum. (ii) a significant group difference between the improvers and decliners of PF over 24-months and their baseline WMM. (iii) no significant difference in longitudinal WM microstructural changes between the improvers and decliners in PF over 24-months.

These results are consistent with a prior study of a 12-month intervention in cognitively normal older adults which also did not find any longitudinal group-level difference due to intervention in WMM (FA, AxD, or RD; Voss et al., 2013). However, that study did find an association between percentage change in a composite score of aerobic fitness and change in FA and found that greater percentage change in fitness was associated with significant increases in prefrontal, parietal and temporal FA within the exercise group, with no significant relationships detected within the control group.

Prior studies have explored the association of higher levels of PA (self-reported or objective measures) and improvement in WMM. Self-reported higher levels of PA have been associated with higher FA in cognitively normal adults over 3 years (Gow et al., 2012; Köhncke et al., 2016). Another study (Best et al., 2017) using objective measures of PA showed that PA levels change over longer periods of aging and may be an important contributor to cognitive and neural protection. Furthermore, the researchers showed that maintaining walking duration over a decade predicted smaller increases in global gray matter MD and WM AxD (*p* < 0.01). At present no other literature exploring the association between objective measures of PF and WMM is available to allow direct comparison with our results.

Irrespective of the specific PF test taken, we found no longitudinal WMM measures (CST and corpus-callosum) associated with PF performance over 24-months. However, if we confine ourselves to the baseline microstructural changes, our results indicate that the association of baseline WMM changes with PF performance over 24-months was highly dependent on the type of PF test chosen. In the cardiovascular fitness test (6-min walk), no significant association with WM structural changes was found. For the mobility/leg strength tests [sit-to-stand and timed-up and go (TUG)], a higher performance in PF was associated with a higher baseline FA in the bilateral CST and simultaneous decreases in AxD. Greater improvement in the TUG test was also significantly associated with a reduced baseline MD of the corpus-callosum. This suggests improvers exhibited more tightly bundled and structured compact fibers compared with decliners (indicated by increased FA in CST) on their baseline scan. The negative association in AxD measures may represent reduced axonal damage in the improvers. For the corpus-callosum, a simultaneous lower MD in these improvers compared to decliners indicates possible cell membrane density changes (Stebbins and Murphy, 2009).

There are two anomalies in our findings that are difficult to explain. For the upper limb strength tests (dominant and non-dominant grip), there are significant results for lower FA in CST of improvers and reduced AxD—it is unclear why improvers in these grip strength tests showed poorer FA values at baseline other than the fact there is more capacity for FA to increase over the 24 months if baseline values were initially low. Another ambiguity regarding the improvers is the right corticospinal tract revealed increased RD in the non-dominant grip test but decreased RD in the dominant grip test. Given RD is often used as a surrogate proxy for the level of demyelination, it is not immediately apparent why these two fitness tests showed opposite behavior in this study. The variability and specificity of these DTI metrics and how they relate to WM pathology is not fully understood yet (Jones et al., 2013).

A previous study (Herting et al., 2014) with adolescent males has shown that significant group differences in CST and the corpus callosum exist between high-fit youth and low-fit peers. It also showed that aerobic fitness was negatively related to FA in the left corticospinal tract. Another study demonstrated that a 6-month aerobic exercise intervention increased in WM volume of the anterior corpus-callosum in physically active older adults (Colcombe et al., 2006). A case study of a world-famous nonagenarian track-and-field athlete (90–95 years), showed higher FA in the genu of the corpus callosum compared to a reference sample of low active women (age 60–78 years; Burzynska et al., 2016).

The corpus callosum plays an integral role in cognitive, motor, and sensory information between the left and right hemispheres of the brain (Sampaio-Baptista and Johansen-Berg, 2017). There is some evidence that an increase in fitness is associated with an increase in α-neurons caused by increased corticospinal tract excitability in addition to adaptive changes in the primary motor cortex, and/or dormant corticospinal connections (Hassanlouei et al., 2017). Rodent studies (Sampaio-Baptista and Johansen-Berg, 2017) suggest that PA promotes oligodendrocyte precursor cell proliferation and differentiation that contributes to WMM measures.

Long-term intervention studies and studies exploring the objective PF measures on WMM in MCI/SMC participants are scarce. A strength of this study is that we studied a larger cohort compared with any long-term PA intervention study previously reported.

One limitation of this study is that we have used four physical performance measures of cardiovascular, mobility, leg strength, and grip strength but have not examined other parameters such as body composition. Another drawback of our study is the relatively low sample size of participants in each group divided by PF measure which does not allow for detailed analysis exploring the interaction of WMM measures with vascular risk factors. The voxel-wise analysis explores only group differences between PF and WMM, as this study was not designed to explore the causation of PF and WMM on cognitive change. We incorporated a LPM to remove the WM lesions to ensure the WMM measures originated from “normal-appearing” WM. By including this LPM mask, spurious associations between fitness tests and WMM in the CST and corpus-callosum were avoided. The output tracts were identified using available atlases, which can be limited by the spatial normalization steps, even though we created a study-specific template to improve this normalization. Future studies should explore advanced multi-compartment models to analyze the diffusion data to infer more specific information about the microstructure.

## Conclusion

In this cohort of participants with SMC, MCI, and vascular risk factors, baseline WM microstructural measures of bilateral CST and corpus-callosum were significantly associated with PF performance over 24 months. The longitudinal WMM measures did not play any role in the physical performance in individuals at risk of Alzheimer’s disease with vascular risk factors. These results indicate that such measures might be used as indicators for recruitment into a personalized PA intervention that would likely have a positive impact on the life of individuals at risk of Alzheimer’s disease. Further research is needed to clarify how WMM measures in adults at risk of dementia may influence their capacity to improve their fitness.

## Data Availability Statement

The data was acquired as part of a clinical trial (AIBLActive—Australia New Zealand Clinical Trial Registry ACTRN 12611000612910). This manuscript is a secondary analysis of the data and does not report on the primary outcome, which has been published in Venkatraman et al. (2020). The datasets used and/or analyzed during the current study are available from the corresponding author on reasonable request.

## Ethics Statement

The studies involving human participants were reviewed and approved by the Melbourne Health Human Research Ethics Committee. The patients/participants provided their written informed consent to participate in this study.

## Author Contributions

VKV and CES: study concept or design, drafting/revising the manuscript, analysis or interpretation of data, statistical analysis, and acquisition of data. KC, KE, PP, MS, VLV, ML, and EC: study concept or design, drafting/revising the manuscript, analysis of the data and acquisition of data. EC, DA, CS, CR, and CM: study concept or design, drafting/revising the manuscript, and study funding. NL and PD: study concept or design, drafting/revising the manuscript, accept responsibility for conduct of research and final approval, study funding, and study supervision.

## Conflict of Interest

CS has provided clinical consultancy and been on scientific advisory committees for the Australian Commonwealth Scientific and Industrial Research Organization, Alzheimer’s Australia, University of Melbourne and other relationships which are subject to confidentiality clauses. She has received funding from Pfizer, Merck, Bayer and GE. She may accrue revenues from patent in pharmacogenomics prediction of seizure recurrence. CR has received research grants from Biogen, Abbvie, Actinogen. The remaining authors declare that the research was conducted in the absence of any commercial or financial relationships that could be construed as a potential conflict of interest.
